# VGF AQEE- and GGEE-peptides differentiate between dementia types

**DOI:** 10.1007/s00415-025-13441-1

**Published:** 2025-11-04

**Authors:** B. Noli, B. Muqaku, M. Gouda, AL. Manai, M. Nagl, S. Anderl-Straub, L. Werner, M. Otto, C. E. Teunissen, P. Oeckl, C. Cocco

**Affiliations:** 1 Department of Biomedical Sciences, University of Cagliari, Cagliari, Italy; 2 Neurochemistry Laboratory, Department of Clinical Chemistry, Amsterdam University Medical Centers (UMC), Amsterdam, the Netherlands; 3 Department of Neurology, Ulm University Hospital, Ulm, Germany; 4 German Center for Neurodegenerative Diseases (DZNE) Ulm, Ulm, Germany; 5 Department of Neurology, Martin-Luther-University Halle-Wittenberg, Halle (Saale), Germany

**Keywords:** Lewy Body dementia, Alzheimer´s disease, VGF, Neuroprotein, Biomarker, Cerebrospinal fluid

## Abstract

Alzheimer’s disease (AD) and dementia with Lewy bodies (DLB) are neurodegenerative disorders with overlapping clinical features, making differential diagnosis challenging. The AQEE and GGEE peptides, derived from the proVGF neuroprotein, have emerged as potential cerebrospinal fluid (CSF) biomarkers for dementia. Indeed, we previously observed a reduction in AQEE-10 levels using selected reaction monitoring (SRM) and GGEE levels using enzyme-linked immunosorbent assay (ELISA) in a cohort of DLB patients compared to both controls and AD patients. To better investigate the diagnostic utility of these peptides, we analyzed CSF samples from both the original cohort and a newly recruited cohort. The new cohort (cohort 1) included patients, from Ulm University Hospital, with Parkinson’s disease dementia (PDD) and DLB (combined as PDD/DLB; *n* = 18), and AD (*n* = 19). The previously analyzed cohort (cohort 2), from the Amsterdam University Medical Center, included DLB (*n* = 44), AD (*n* = 20), and cognitively healthy controls (*n* = 22). AQEE-10 levels were quantified by multiple reaction monitoring (MRM) in cohort 1 and by ELISA in both cohorts. GGEE levels were measured by ELISA in cohort 1 to corroborate and extend previous findings. MRM-based analysis revealed a significant reduction of AQEE-10 levels in DLB compared to both controls and AD (*p* < 0.05; ROC-AUC: 78% and 82%, respectively). This finding was confirmed by ELISA, for both AQEE-10 and GGEE peptide levels, along with a positive correlation between their concentrations. These results support AQEE-10 and GGEE as promising peptide biomarkers for distinguishing DLB from other dementia.

**Supplementary Information:**

The online version contains supplementary material available at 10.1007/s00415-025-13441-1.

## Introduction

Alzheimer’s disease (AD) and dementia with Lewy bodies (DLB) are among the most common forms of neurodegenerative dementia. Although each condition is characterized by distinct pathological features, their clinical manifestations often overlap, making differential diagnosis challenging [1]. This highlights the urgent need for more accurate and reliable diagnostic tools capable of distinguishing between dementia subtypes. Neuropeptides, which are small protein-like molecules secreted by neurons, have emerged as promising fluid-based biomarkers for neurodegenerative diseases. Once secreted, these peptides can diffuse into the bloodstream or cerebrospinal fluid (CSF), making them accessible for diagnostic analysis [2]. Among these, proVGF—a precursor protein stored in dense-core vesicles within neurons—is processed into multiple bioactive peptides of varying molecular weights [3]. Peptides derived from VGF, particularly those containing AQEE and GGEE motifs, have been proposed as candidate biomarkers for neurodegenerative dementias. Our earlier studies showed reduced levels of proVGF C-terminal peptides—potentially including AQEE-30—in post-mortem brain cortices from AD patients compared to cognitively normal controls [4]. Similarly, lower concentrations of AQEE-10, a truncated form of AQEE-30, were detected in CSF samples from AD patients relative to non-demented individuals [5]. The GGEE peptide has also been implicated in AD, with decreased CSF levels in affected individuals compared to healthy controls [6]. These findings indicate that levels of both AQEE and GGEE peptides are reduced in AD. However, in our previous studies, we demonstrated that DLB patients could be reliably distinguished from both AD patients and controls by a significant decrease in the levels of AQEE-10 measured using selective mass spectrometry (SMR) and GGEE peptides (quantified using enzyme-linked immunosorbent assay) [7–9]. It is important to note, however, that the SMR-based measurement of AQEE-10 has not yet been validated by an independent analytical method. Regarding their biological function, AQEE-30 has been implicated in synaptic plasticity [10], neuroprotection [11], and nociceptive processing within the spinal cord [12]. In contrast, the role of GGEE peptides in neuronal function remains largely unclear. Given the proposed neuronal functions of the AQEE peptides, our first aim was to more specifically assess AQEE-10 as a potential biomarker for DLB. Our second aim was to strengthen the evidence for GGEE as a diagnostic biomarker by expanding the original study cohort. To achieve these goals, we employed two complementary techniques for AQEE-10 quantification: multiple reaction monitoring (MRM), one of the most sensitive methods for peptide measurement, and enzyme-linked immunosorbent assay (ELISA). In parallel, we measured GGEE levels using ELISA and evaluated their correlation with AQEE concentrations.

## Materials and methods

### Subjects involved in the study

This study includes two independent cohorts of subjects (Table 1). CSF samples were collected during the diagnostic workup of patients at the Department of Neurology, Ulm University Hospital, Ulm, Germany (Cohort 1), and at Amsterdam University Medical Center (AUMC), Amsterdam, Netherlands (Cohort 2). Cohort 1 comprised patients diagnosed with AD (*n* = 19), and DLB/Parkinson's disease dementia (grouped as DLB/PDD; *n* = 18). It also included age-matched control subjects (*n* = 27) who did not present with neurodegenerative conditions but underwent CSF collection to exclude neuroinflammatory disorders. Control diagnoses included facial palsy (*n* = 11), tension headache (*n* = 6), trochlear paresis (*n* = 2), intoxication, physical and mental stress/prostate carcinoma, migraine, ocular myositis, pansinusitis, polyneuropathy/restless leg syndrome, right leg pain syndrome, and vertigo. Patients in the dementia groups were diagnosed according to established clinical criteria [13–16]. CSF levels of total tau, phosphorylated tau at threonine 181 (pTau181), and amyloid-beta 42 (Aβ42) were measured using ELISA kits from Fujirebio Germany GmbH (Hannover, Germany) during routine clinical evaluation. Only patients with probable DLB were included. The study was approved by the ethics committee of Ulm University (approval no. 20/10). Cohort 2 included CSF samples from patients with AD (*n* = 20) and DLB (*n* = 44), as well as age-matched non-neurodegenerative controls (*n* = 22). CSF concentrations of tau, pTau181, Aβ42, and α-synuclein were assessed using ELISA kits as part of standard clinical procedures. Demographic clinical characteristics and approvement by the ethics committee of cohort 2 were previously described [7]. All CSF samples were obtained via lumbar puncture, centrifuged, and stored within 2 h at − 80 °C in polypropylene tubes.

**Table 1:** Patient characteristics

	Controls	AD	PDD/DLB
COHORT 1 (*n* = 82)			
Patient (*n*)	27	19	18
Female (*n*, %)	9 (33%)	7 (37%)	5 (28%)
Age	69 [48–82]	74 [65–81]	73 [62–82]
Aβ1-42 (pg/mL)	1290 [763–1772]	438 [287–689]	613 [369–1154]
Tau (pg/mL)	286 [217–675]	812 [421–1773]	364 [183–903]
p-Tau (pg/mL)	37 [20–79]	72 [20–236]	61 [44–110]
COHORT 2 (*n* = 86)			
Patient (*n*)	22	20	44
Female (*n*, %)	4 (18%)	2 (10%)	5 (11%)
Age	63 [55–74]	65 [54–76]	67 [54–78]
Aβ1-42 (pg/mL)	1040 [785–1335]	586 [440–700]	780 [436–1404]
Tau (pg/mL)	194 [79–355]	596 [314–1776]	292 [68–914]
p-Tau (pg/mL)	39 [19–52]	88 [57–252]	47 [16–158]
α-synuclein (pg/mL)	1465 [697–2717]		1805 [798–3524]

Controls are patients without dementia or other neurodegenerative diseases; AD, Alzheimer's disease; PDD, Parkinson's disease dementia; DLB, dementia with Lewy bodies; Aβ1-42, amyloid β-peptide (1–42); p-Tau, phosphorylated Tau; α-synuclein, alpha-synuclein. Data are presented as median [min–max] or *n* (%); pg/mL: picograms/milliliters

### MRM analysis of AQEE-10

The MRM method for quantifying the AQEE-10 in CSF has been described previously [7, 17] In brief, CSF sample preparation involved reduction and alkylation, followed by overnight enzymatic digestion at 37 °C using a trypsin/LysC mixture. The resulting peptides were fractionated using strong cation exchange (SCX) STAGE Tips. Peptide separation was performed on an Eksigent MicroLC200 chromatographic system, and analysis was carried out on a QTRAP 6500 mass spectrometer (AB Sciex, Darmstadt, Germany). The AQEE-10 peptide (VGF586–595) was quantified (in cohort 1) using MRM with the following transitions: for the endogenous peptide, 581.3 → 962.4 (y8), 581.3 → 833.4 (y7), and 581.3 → 704.3 (y6); and for the isotopically labeled standard (heavy peptide), 586.3 → 972.4 (y8), 586.3 → 843.4 (y7), and 586.3 → 714.3 (y6) (Supplemental materials Table S1). The performance characteristics of the MRM method are summarized in Table 2. All MRM data were processed and evaluated using Skyline software [18] and results were reported as abundance ratios between endogenous peptides and their corresponding isotopically labeled internal standards (light/heavy, L/H ratio).

**Table 2:** MRM assay performance

Protein name	Stability test (*n* = 2)	Dilution linearity (*n* = 2)	Intra-assay variation (*n* = 5)
AQEE	2 h RT: 100.7–106.7	1 to 2: 88.8—103.6	2.2
	1 cycle: 94.1–104.9	1 to 4: 81.8—88.3	
	3 cycles: 95.4–97.7	1 to 8: 85.9—90.6	
	5 cycles:102.3–97.4		

2 h RT—incubated for 2 h at room temperature; 1 cycle—one freeze–thaw cycle; 3 cycles—three freeze–thaw cycles; 5 cycles—five freeze–thaw cycles. n: number of replicates

### Competitive ELISA

For the AQEE immunoassay, a polyclonal anti-AQEE antibody was generated in rabbits against the AQEE-10 peptide (VGF586–595), conjugated to bovine thyroglobulin via an additional C-terminal cysteine. The antibody was affinity-purified by incubation with the immunogen covalently immobilized on SulfoLink Coupling Resin (Thermo Fisher Scientific), followed by extensive washing with phosphate-buffered saline (PBS, 0.5 M), and elution was performed using 1 M glycine–HCl buffer (pH 2.5). Details of the AQEE antibody production and assay validation have been previously reported [19]. The GGEE (VGF373-417) immunoassay was performed as previously described [7]. For ELISA measurements, microtiter plates were coated with the respective peptides (AQEE-10 or GGEE-9) diluted in carbonate/bicarbonate buffer (pH 9.6), then blocked using PBS-Tween 20 (0.01 mol/L phosphate buffer, pH 7.2–7.4, 0.15 mol/L NaCl, 0.5 g/L Tween 20) supplemented with normal donkey serum (90 mL/L), aprotinin (20 nmol/L), and ethylenediaminetetraacetic acid (EDTA: 1 g/L). Plates were incubated at room temperature for 3 h with a mixture of the primary antibody (diluted in blocking buffer) and serial dilutions of either the standard peptide (0.005–500 pmol/mL) or the samples. Following incubation, plates were washed and treated sequentially with a biotinylated secondary antibody (1 h, 1:10,000 dilution; Jackson ImmunoResearch, West Grove, PA, USA), a streptavidin–peroxidase conjugate (30 min, 1:10,000; Biospa, Milan, Italy), and tetramethylbenzidine (TMB; X-tra, Kem-En-Tec, Taastrup, Denmark). The enzymatic reaction was stopped with 1 M HCl, and absorbance was measured at 450 nm using a multilabel plate reader (Chameleon, Hidex, Turku, Finland). Antibody dilutions were 1:10,000 for GGEE assay and 1:8,000 for AQEE assay.

### Statistical analysis

Statistical analyses were performed using GraphPad Prism v.8 (GraphPad Software, San Diego, CA, USA), R software v. 4.1.0, and StatistiXL Software (www.statistixl.com). For MRM and ELISA data, the normality of distribution was tested with Shapiro–Wilk, and the presence of outliers with the Grubbs test. CSF levels of VGF peptides were not normally distributed; nonparametric tests were used for any of the analyses. Groups were compared using the Kruskal–Wallis test, followed by Dunn’s post hoc test with Bonferroni correction for multiple comparisons. Correlation analyses were performed using Spearman’s rank correlation coefficient. Receiver operating characteristic (ROC) curves were generated in R v. 4.1.0 by using the packages pROC and nnet. A p-value < 0.05 was regarded as statistically significant.

## Results

### AQEE-MRM, AQEE-ELISA, and GGEE-ELISA in cohort 1

**Fig. 1:**
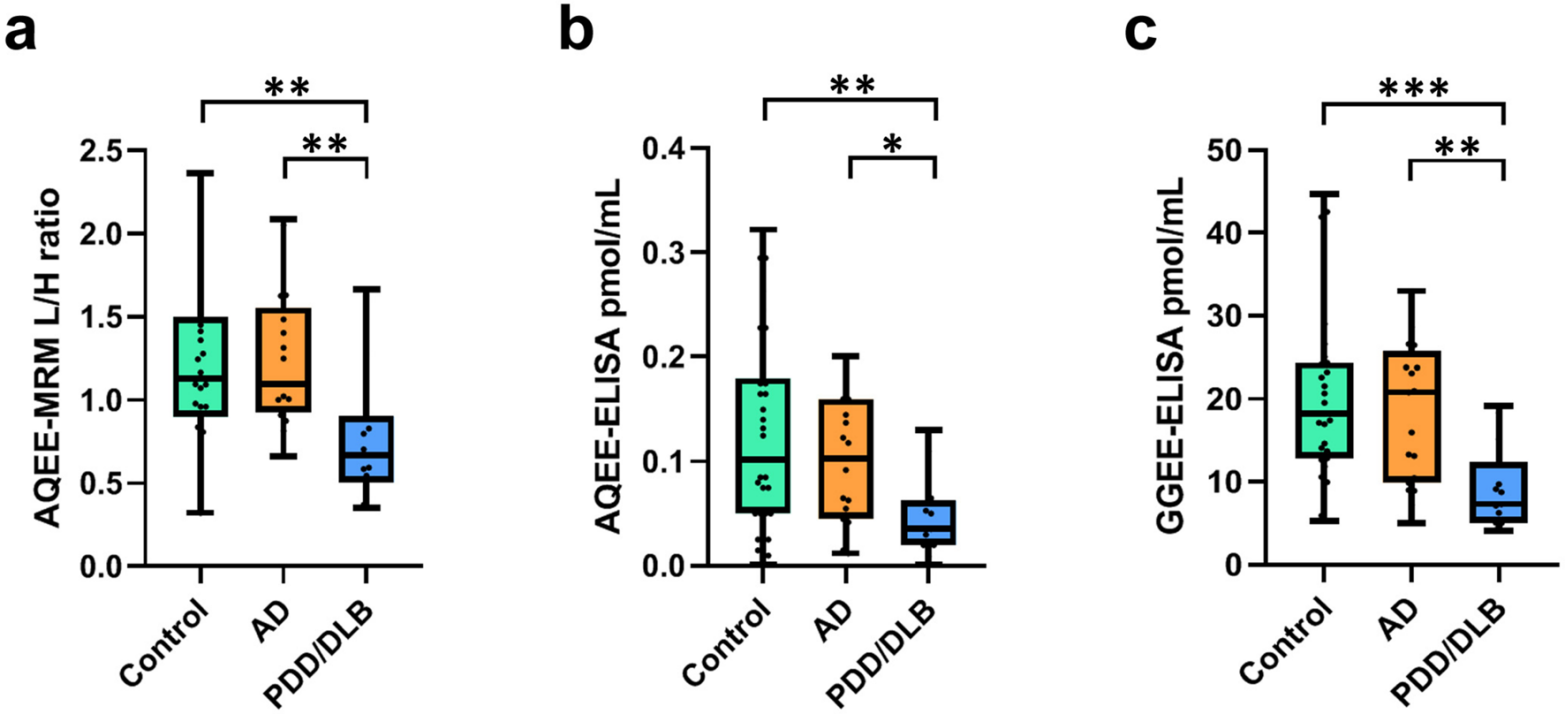
AQEE and GGEE peptide levels in cohort 1. **a** AQEE levels measured by MRM in cohort 1 (data available for 24 controls, 17 AD, and 15 PDD/DLB patients). Boxplots indicate the median (line) and the range (whiskers showing minimum and maximum values). Boxplots represent the light-to-heavy (L/H) peptide ratio obtained from MRM analyses. **b** AQEE levels measured by ELISA in cohorts 1 (data available for 27 controls, 18 AD, and 13 PDD/DLB patients). **c** GGEE levels measured by ELISA in cohort 1 (data available for 27 controls, 19 AD and 13 PDD/DLB patients). Boxplots indicate the median (line) and the range (whiskers showing minimum and maximum values). Statistical comparisons were performed using the Kruskal–Wallis test and Dunn’s multiple comparisons test. AD, Alzheimer’s disease; PDD/DLB, Parkinson’s disease dementia/dementia with Lewy bodies; Control, non-neurodegenerative controls. Pmol/mL, picomoles/milliliters. * *p* < 0.05; ** *p* < 0.005; *** *p* < 0.0005

The validated MRM method was applied to quantify AQEE-10 levels in cohort 1 (Fig. 1a), which included 27 control subjects, 19 patients with AD, and 18 with PDD/DLB. All samples were analyzed in a single analytical run with an intra-assay CV in QC (quality control) samples of 7.4% (*n* = 6). Data were unavailable (due to technical reasons or sensitivity) for 13 individuals: 3 controls, 2 AD, and 3 PDD/DLB patients. AQEE-10 levels were significantly reduced in the PDD/DLB group (0.67 [0.5–0.9] L/H ratio, median [interquartile range]) compared to both controls (1.13 [0.9–1.5]; *p* = 0.009) and AD patients (1.10 [0.9–1.6]; *p* = 0.009). PDD/DLB patients exhibited significantly lower AQEE-10 levels compared to controls, and these patients had also less levels of AQEE-10 than AD patients. The cohort 1 was also analyzed for both AQEE and GGEE levels (Fig. 1b, c). AQEE measurements were unavailable for 6 subjects (1 AD, and 5 PDD/DLB), while GGEE data were missing for 4 PDD/DLB patients Unavailable/missing values were statistical outliers. AQEE and GGEE peptide levels were significantly lower in patients with PDD/DLB (AQEE: 0.01 [0.008–0.05] pmol/mL, median [interquartile range]; GGEE: 7.3 [5.1–12.4] pmol/mL) compared to controls (AQEE: 0.13 [0.05–0.19] pmol/mL *p* = 0.003; GGEE: 18.3 [12.8–24.4] pmol/mL, *p* = 0.0007) and AD patients (AQEE: 0.12 [0.07–0.17] pmol/mL, *p* = 0.04; GGEE: 20.8 [9.9–25.7] pmol/mL, *p* = 0.006). In conclusion, PDD/DLB patients exhibited significantly reduced levels of both AQEE and GGEE peptides compared to cognitively normal controls and AD patients, supporting the findings obtained through MRM.

### AQEE-ELISA in cohort 2

**Fig. 2:**
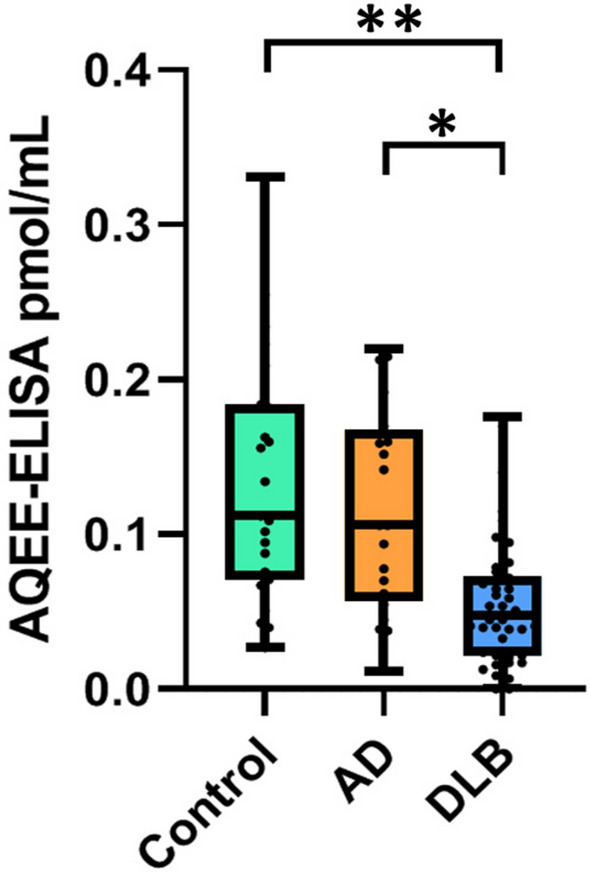
AQEE-ELISA levels in cohort 2. AQEE levels measured by ELISA in cohort 2 (data available for 22 controls, 20 AD and 44 DLB). Boxplots indicate the median (line) and the range (whiskers showing minimum and maximum values). Statistical comparisons were performed using the Kruskal–Wallis test and Dunn’s multiple comparisons test. AD, Alzheimer’s disease; DLB, dementia with Lewy bodies; Control: non-neurodegenerative controls. Pmol/mL, picomoles/milliliters. * *p* < 0.05; ** *p* < 0.005; *** *p* < 0.0005

Since cohort 2 was alredy used for AQEE-SMR and GGEE-ELISA analysis, this cohort underwent the AQEE- ELISA only (Fig. 2). The AQEE peptide levels were significantly lower in patients with PDD/DLB (0.05 [0.02–0.08] pmol/mL) compared to controls (0.11 [0.07–0.18] pmol/mL, *p* = 0.009) and AD patients (0.1 [0.06–0.17] pmol/mL, *p* = 0.04). In conclusion, PDD/DLB showed significantly decreased levels of AQEE peptides relative to cognitively normal controls and AD patients.

### Correlation analyses

The levels of the two VGF-derived peptides, measured using either ELISA or MRM, were correlated with each other within each diagnostic group. Additionally, correlations were assessed between each VGF peptide and misfolded protein biomarkers—including pTau, total Tau, Aβ1-42, and α-synuclein—within the patient groups (Table 3). Correlation results for all patients combined are also presented in supplementary materials, Figs. S1 and S2 for cohorts 1 and 2, respectively. The analysis of combined patients revealed a significant positive correlation between the two peptides measured by ELISA, as well as between AQEE-MRM and both GGEE and AQEE levels measured by ELISA (supplementary materials, Fig. S1). Furthermore, in cohort 2, AQEE levels measured by ELISA were positively correlated with Tau and pTau, while a strong correlation between AQEE (ELISA) and α-synuclein was observed in patients with PDD/DLB (supplementary materials, Fig. S2).

**Table 3:** Correlations between CSF biomarkers

	Total group		Controls		AD		PDD/DLB	
	ρ	*p*	ρ	*p*	ρ	*p*	ρ	*p*
Cohort 1								
AQEE-ELISA *vs* AQEE-MRM	0.41	**0.003**	0.26	0.22	0.01	0.98	0.53	0.12
GGEE-ELISA *vs* AQEE-MRM	0.88	1.6*10^**–9**^	0.87	1.2*10^**–12**^	0.77	**0.0006**	0.95	1.1*10^**–20**^
GGEE-ELISA *vs* AQEE-ELISA	0.50	8.4*10^**–5**^	0.24	0.24	0.38	0.11	0.58	**0.038**
AQEE-MRM *vs* Tau	0.33	0.059	0.74	**0.047**	0.31	0.23	0.76	**0.037**
AQEE-MRM *vs* p-Tau	0.06	0.75	0.31	0.46	0.19	0.47	-0.5	1.0
AQEE-MRM *vs* Aβ 1–42	0.027	0.88	0.31	0.46	-0.17	0.51	0.14	0.74
AQEE-ELISA *vs* Tau	0.15	0.36	-0.34	0.38	0.13	0.6	0.71	**0.027**
AQEE-ELISA *vs* p-Tau	-0.002	0.99	0.024	0.97	0.19	0.45	0.66	0.23
AQEE-ELISA *vs* Aβ 1–42	0.13	0.44	-0.18	0.65	0.20	0.43	-0.42	0.24
GGEE-ELISA *vs* Tau	0.38	**0.019**	0.67	0.059	0.30	0.21	0.79	**0.009**
GGEE-ELISA *vs* p-Tau	0.26	0.15	0.33	0.38	0.31	0.21	0.7	0.23
GGEE-ELISA *vs* Aβ 1–42	0.17	0.31	0.63	0.08	0.14	0.57	0.08	0.83
Cohort 2								
AQEE-ELISA *vs* Tau	0.22	**0.049**	0.7	**0.0007**	0.29	0.23	0.46	**0.002**
AQEE-ELISA *vs* p-Tau	0.34	**0.001**	0.62	**0.003**	0.53	**0.017**	0.54	**0.0002**
AQEE-ELISA *vs* Aβ1-42	0.04	0.69	0.44	**0.046**	0.077	0.75	-0.08	0.63
AQEE-ELISA *vs* α-Syn	0.44	**0.0003**	0.72	**0.001**	-	-	0.63	9.4*10^**–6**^

Associations were assessed with Spearman correlation coefficient (ρ), *p*-values in bold are < 0.05. AD: Alzheimer's disease; PDD: Parkinson's disease dementia; DLB: dementia with Lewy bodies; bvFTD: behavioral variant frontotemporal dementia; Aβ1-42**:** amyloid β-peptide (1–42); p-Tau: phosphorylated Tau; α-syn: alpha-synuclein

### Receiver operating characteristic curve analyses

**Fig. 3:**
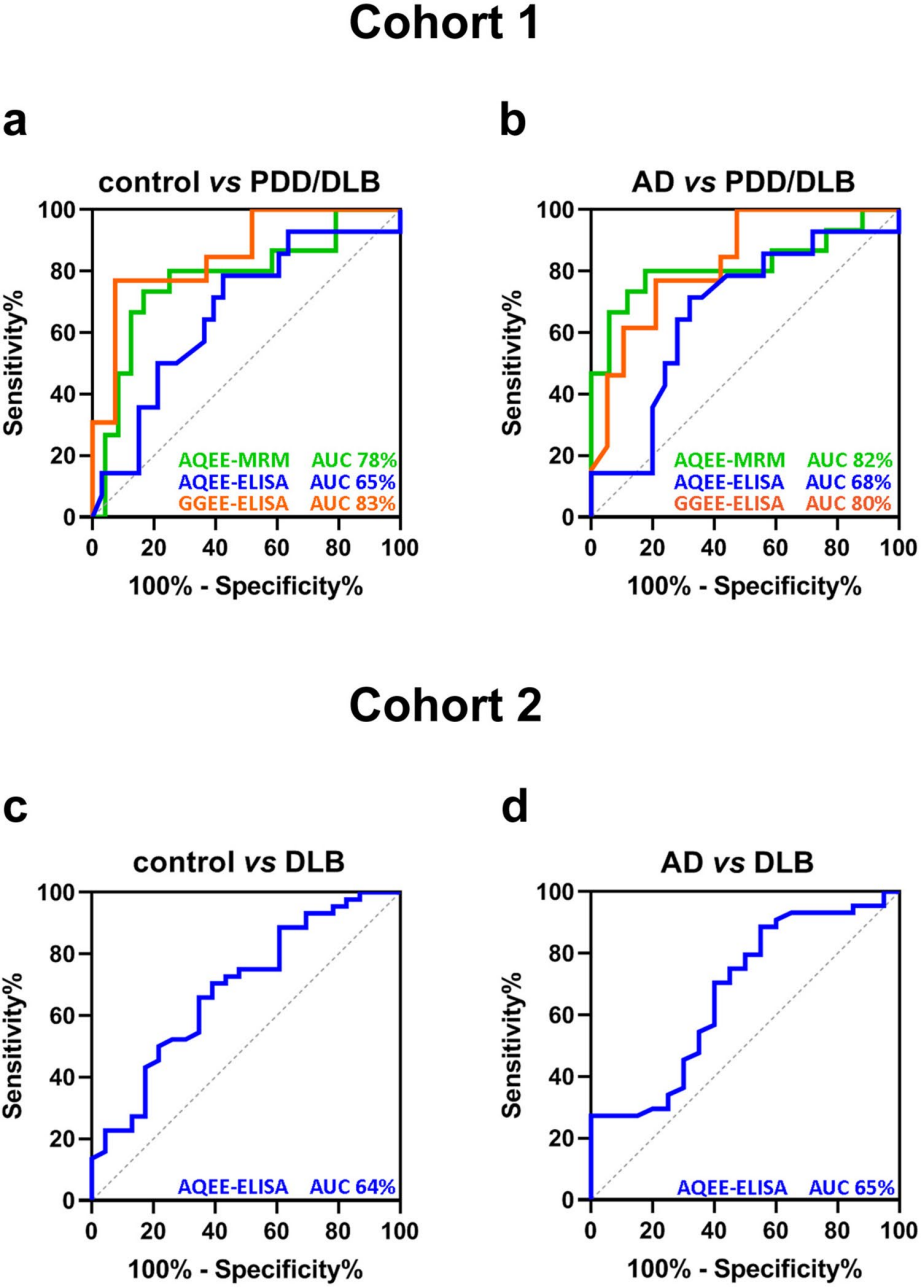
ROC curve analyses. Receiver operating characteristic** (**ROC) curves were generated using cohort 1 and cohort 2. The area under the curve (AUC) is shown for each comparison. DLB: dementia with Lewy bodies; PDD: Parkinson’s disease dementia; AD: Alzheimer’s disease

To assess whether AQEE and GGEE peptide levels could effectively differentiate PDD/DLB patients from healthy controls and other dementia subtypes (AD), receiver operating characteristic (ROC) curve analyses were conducted for cohort 1 (Fig. 3a,b). For Cohort 2, ROC curve analysis was performed using only AQEE levels (Fig. 3c,d), as data for GGEE and SMR-based measurements had already been published previously [7]. The highest area under the curve (AUC) value was observed when distinguishing AD from PDD/DLB using AQEE-MRM, with an AUC of 0.82 and when comparing controls with PDD/DLB using GGEE-ELISA (AUC = 0.83).

## Discussion

In the present study, our main finding is that, among the dementia subtypes investigated, patients with PDD/DLB exhibited significantly reduced CSF levels of the AQEE-10 peptide compared to cognitively normal controls and AD patients, as measured by both MRM and competitive ELISA using an antibody specifically produced against the AQEE-10. Furthermore, using ELISA, we identified a significant correlation between GGEE and AQEE levels, confirming our previous observations of reduced GGEE in PDD/DLB patients [7], In addition to the differences observed between PDD/DLB patients and controls, we also detected distinctions among the dementia subtypes (AD and PDD/DLB). Since the exogenous AQEE-30 peptide acutely increases synaptic charge in a dose-dependent manner [10], the reduction in AQEE levels we observed may reflect a corresponding decrease in this synaptic activity, although targeted studies are needed to elucidate AQEE-specific function in the context of dementia. Previous studies have reported reduced levels of AQEE [5] and GGEE [5, 6] peptides in the CSF of AD patients using mass spectrometry. In contrast, our results did not show reduced AQEE or GGEE levels in AD patients relative to controls. This discrepancy may be attributable to differences in clinical and diagnostic characteristics across cohorts. Notably, our patient cohort was diagnosed according to established clinical criteria [13–16] but also underwent comprehensive CSF biomarker profiling—including Tau, pTau181, and Aβ42 levels as shown in Tabe 1. This level of biomarker confirmation was often lacking in the previous mentioned studies in which the full panel of biomarkers that we analysed, were not measured but rather only one of these [5, 6]. The correlation between our peptides and alpha-synuclein in PDD/DLB was expected, while the ones with pTau and Tau may suggest that VGF peptides’ expression might be linked to specific neurodegenerative processes, particularly those involving tau pathology. However, the biological significance of these correlations remains unclear at this stage and must be interpreted with caution. Our findings are strengthened by the use of two orthogonal analytical methods (MRM and ELISA) and by replication in two independent cohorts. Indeed, cohort 2 was previously used in a published study [7] for SRM, using AQEE as a standard, as well as for GGEE-based ELISA. In that study, AQEE levels were not assessed by ELISA—only the GGEE-ELISA assay was performed. Interestingly, the VGF levels obtained via both SRM and GGEE-ELISA in that previous study were comparable to those observed in the current study using cohort 1 with MRM and GGEE-ELISA, with similarly decreased levels in DLB compared to AD and control groups. Moreover, the AQEE levels measured in cohort 2 in the present study showed a reduction similar to that observed in cohort 1. In conclusion, these findings support the comparability of cohort 1 and cohort 2 (supplementary materials, Table 2). However, the exploratory nature of this study presents certain limitations, the most significant being the sample size. Furthermore, future studies should employ more sensitive and specific immunoassays capable of reliably distinguishing AQEE-10 from the other VGF-derived peptides given that several AQEE peptides may exist beyond that we identified (i.e. AQEE-30 or the proVGF itself) because the anti-AQEE antibody potentially recognizes all peptides containing AQEE sequence. Indeed, the scenario of the modulation of specific VGF peptides under pathological conditions appears highly complex. For example, a recent study in multiple sclerosis (MS) patients reported elevated serum AQEE levels compared to healthy controls, whereas GGEE levels remained unchanged [20]. These findings suggest that VGF-derived peptides may be differentially regulated even within a single pathological condition. This complexity is further exemplified in neurodegenerative diseases such as amyotrophic lateral sclerosis (ALS), where patients in early stages exhibit elevated plasma levels of certain VGF-derived "NERP peptides" [21] while others—such as TLQP peptides—are decreased in both early and late disease stages, alongside further reductions in advanced stages of peptides derived from the C-terminal region of proVGF [22]. In conclusion, our consistent findings using highly specific MRM, validated by independent ELISA measurements, highlight AQEE as a novel and promising VGF-derived biomarker for identifying PDD/DLB and distinguishing it from AD. Given that GGEE was also validated as a biomarker for DLB, we propose that both peptides should be systematically investigated in future large-scale, longitudinal studies. The discovery and validation of such novel diagnostic biomarkers may substantially enhance disease classification and facilitate the development of more personalized therapeutic strategies.

## Supplementary Information

Below is the link to the electronic supplementary material.

Supplementary file1 (JPG 596 KB) Fig. S1 Correlation analysis in cohort 1. Correlation analyses between AQEE and GGEE peptide levels, as well as between each peptide and the core CSF biomarkers, were conducted using all CSF samples from cohort 1. Biomarkers include amyloid-beta (Aβ), Tau and phosphorylated tau (p-Tau). Concentrations are expressed as follows: peptides in picomoles per milliliter (pmol/mL) and proteins in picograms per milliliter (pg/mL)

Supplementary file2 (JPG 223 KB) Fig. S2 Correlation analysis in cohort 2. Correlation analyses between AQEE peptide levels and the core CSF biomarkers, were conducted using all CSF samples from cohort 2. Biomarkers include alpha-synuclein (α-syn), amyloid-beta (Aβ), Tau and phosphorylated tau (p-Tau). Concentrations are expressed as follows: peptides in picomoles per milliliter (pmol/mL) and proteins in picograms per milliliter (pg/mL)

Supplementary file3 (Table 1, XLSX 12 KB)

Supplementary file4 (Table 2, DOCX 14 KB)
