# Supplementary figures and images for: VGF AQEE- and GGEE-peptides differentiate between dementia types
Source: J Neurol. 2025 Nov 4;272(11):745. doi: 10.1007/s00415-025-13441-1 (PMC12586231; doi:10.1007/s00415-025-13441-1)

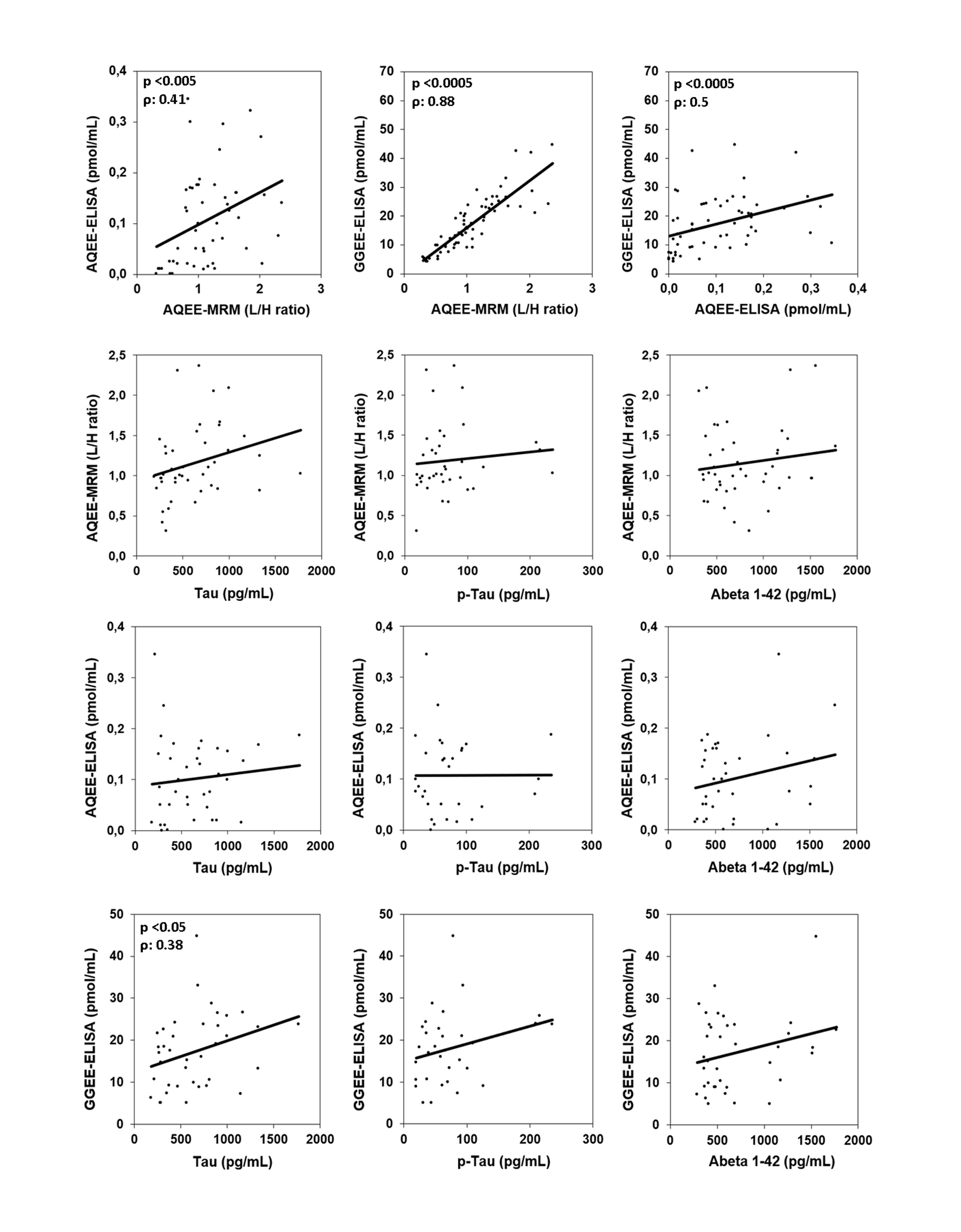

Supplement: Supplementary file 1 — Supplementary file1 (JPG 596 KB) Fig. S1 Correlation analysis in cohort 1. Correlation analyses between AQEE and GGEE peptide levels, as well as between each peptide and the core CSF biomarkers, were conducted using all CSF samples from cohort 1. Biomarkers include amyloid-beta (Aβ), Tau and phosphorylated tau (p-Tau). Concentrations are expressed as follows: peptides in picomoles per milliliter (pmol/mL) and proteins in picograms per milliliter (pg/mL) [file 415_2025_13441_MOESM1_ESM.jpg]

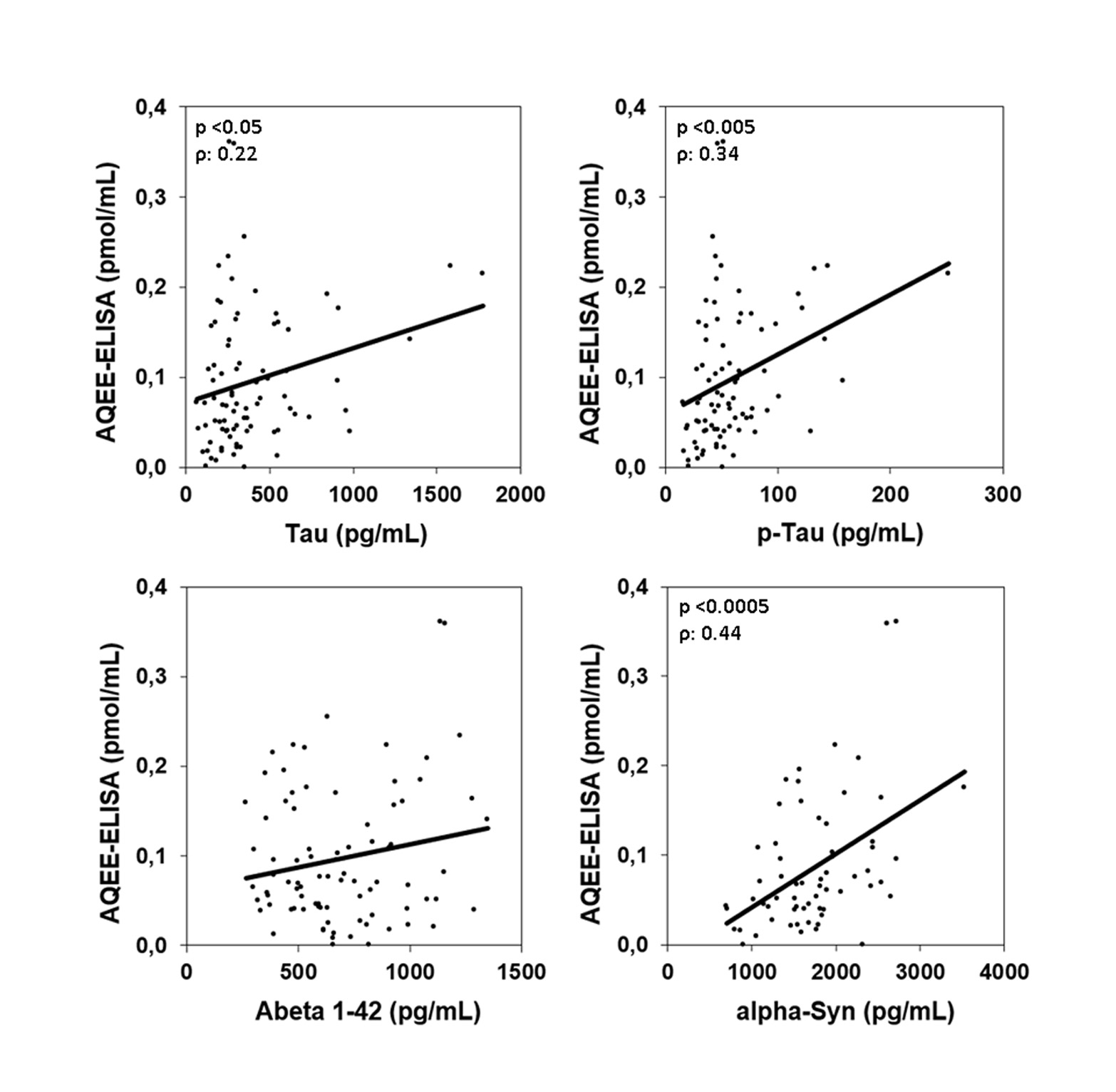

Supplement: Supplementary file 2 — Supplementary file2 (JPG 223 KB) Fig. S2 Correlation analysis in cohort 2. Correlation analyses between AQEE peptide levels and the core CSF biomarkers, were conducted using all CSF samples from cohort 2. Biomarkers include alpha-synuclein (α-syn), amyloid-beta (Aβ), Tau and phosphorylated tau (p-Tau). Concentrations are expressed as follows: peptides in picomoles per milliliter (pmol/mL) and proteins in picograms per milliliter (pg/mL) [file 415_2025_13441_MOESM2_ESM.jpg]
