# Supplementary material for: VGF AQEE- and GGEE-peptides differentiate between dementia types
Source: J Neurol. 2025 Nov 4;272(11):745. doi: 10.1007/s00415-025-13441-1 (PMC12586231; doi:10.1007/s00415-025-13441-1)
Supplement: Supplementary file 4 — Supplementary file4 (Table 2, DOCX 14 KB) [file 415_2025_13441_MOESM4_ESM.docx]

| **Technique** | **Cohort 1** | **Cohort 2** |
| --- | --- | --- |
| **AQEE-SMR** | not performed | reduction in DLB  *vs* controls and AD  [7] |
| **AQEE-MRM** | reduction in PDD/DLB  *vs* controls and AD  (present paper) | not performed |
| **AQEE-ELISA** | reduction in PDD/DLB  *vs* controls and AD  (present paper) | reduction in DLB  *vs* controls and AD  (present paper) |
| **GGEE-ELISA** | Reduction in PDD/DLB  *vs* controls and AD  (present paper) | reduction in DLB  vs controls and AD  [7] |

**Table 2 . Comparison between cohort 1 and cohort 2.**
